# Characterization of Pneumococcal Genes Involved in Bloodstream Invasion in a Mouse Model

**DOI:** 10.1371/journal.pone.0141816

**Published:** 2015-11-05

**Authors:** Layla K. Mahdi, Mark B. Van der Hoek, Esmaeil Ebrahimie, James C. Paton, Abiodun D. Ogunniyi

**Affiliations:** 1 Research Centre for Infectious Diseases, School of Biological Sciences, The University of Adelaide, Adelaide, South Australia, Australia; 2 Adelaide Microarray Centre, The University of Adelaide and SA Pathology, Adelaide, South Australia, Australia; 3 Department of Genetics and Evolution, School of Biological Sciences, The University of Adelaide, Adelaide, South Australia, Australia; Albany Medical College, UNITED STATES

## Abstract

*Streptococcus pneumoniae* (the pneumococcus) continues to account for significant morbidity and mortality worldwide, causing life-threatening diseases such as pneumonia, bacteremia and meningitis, as well as less serious infections such as sinusitis, conjunctivitis and otitis media. Current polysaccharide vaccines are strictly serotype-specific and also drive the emergence of non-vaccine serotype strains. In this study, we used microarray analysis to compare gene expression patterns of either serotype 4 or serotype 6A pneumococci in the nasopharynx and blood of mice, as a model to identify genes involved in invasion of blood in the context of occult bacteremia in humans. In this manner, we identified 26 genes that were significantly up-regulated in the nasopharynx and 36 genes that were significantly up-regulated in the blood that were common to both strains. Gene Ontology classification revealed that transporter and DNA binding (transcription factor) activities constitute the significantly different molecular functional categories for genes up-regulated in the nasopharynx and blood. Targeted mutagenesis of selected genes from both niches and subsequent virulence and pathogenesis studies identified the manganese-dependent superoxide dismutase (SodA) as most likely to be essential for colonization, and the cell wall-associated serine protease (PrtA) as important for invasion of blood. This work extends our previous analyses and suggests that both PrtA and SodA warrant examination in future studies aimed at prevention and/or control of pneumococcal disease.

## Introduction

*Streptococcus pneumoniae* (the pneumococcus) is a formidable human pathogen, responsible for significant morbidity and mortality worldwide. It causes a broad spectrum of diseases ranging from less serious infections such as sinusitis, conjunctivitis and otitis media, to potentially fatal diseases such as pneumonia, bacteraemia and meningitis. The burden of pneumococcal disease is greatest in developing countries, where an estimated 1.1 million children under 5 years of age die each year from pneumonia (approximately 20% of all deaths in this age group), of which *S*. *pneumoniae* is the single commonest cause [1]. The continuing problem of pneumococcal disease is partly attributable to the rate at which this organism is acquiring resistance to multiple antimicrobials and the rapid global spread of highly resistant clones [2]. The problem is being exacerbated by the major shortcomings associated with the current capsular polysaccharide-based vaccines, including cost, strictly serotype-specific protection, incomplete serotype coverage, and replacement carriage and disease caused by non-vaccine serotypes [3].

Concerted global efforts are focused on accelerating the development of alternative pneumococcal vaccine strategies that address the shortcomings of existing approaches, without compromising efficacy. One such approach involves a detailed assessment of pneumococcal proteins that contribute to pathogenesis and are common to all serotypes, their development as vaccine antigens, and an understanding of the mechanism whereby protection might be elicited. The virulence proteins which have received the greatest attention and shown consistent promise as vaccine candidates to date include the thiol-activated toxin pneumolysin (Ply), two choline-binding surface proteins called pneumococcal surface protein A (PspA) and choline-binding protein A (CbpA) (also referred to as PspC or SpsA), iron uptake protein PiuA and various combinations thereof [4–11].

Additional candidate proteins have been identified and appraised for inclusion in multi-component pneumococcal protein vaccine formulations. These include autolysin (LytA) [12], heat-shock protein ClpP [13], neuraminidase A (NanA) [14, 15], pili (RrgA, RrgB, RrgC) [16], the polyhistidine triad (Pht) proteins (particularly PhtD) [6, 17–19], PotD [20], StkP and PcsB [21]. Experimental multivalent protein vaccines are currently being optimized to obtain the best formulation that could confer sufficiently synergistic protection against a wider variety of *S*. *pneumoniae* strains to warrant clinical development as an alternative to existing conjugate vaccines. As part of these activities, we carried out systematic microarray comparisons of gene expression kinetics of two pneumococcal strains (WCH16 [serotype 6A] and WCH43 [serotype 4]) in the nasopharynx, lungs, blood and brain of mice. The analyses yielded a number of niche-specific, up-regulated genes that contribute to pathogenesis, some of which were shown to encode good vaccine candidates [22, 23]. However, direct comparisons of gene expression profiles of these pneumococci between the nasopharynx and blood is yet to be reported, although in early childhood, this direct transition to blood is a complication of pneumococcal carriage [24]. We hypothesized that pneumococcal genes that are consistently up-regulated in the blood relative to nasopharynx are likely to be important for survival and/or virulence, while those that are consistently up-regulated in the nasopharynx relative blood are likely to be genes that are important for colonization. In order to test this hypothesis, we carried out comparison of pneumococcal gene expression patterns between the nasopharynx and blood using existing transcriptomic data derived from the two *S*. *pneumoniae* strains after intranasal challenge of mice.

## Materials and Methods

### Ethics Statement

Outbred 5- to 6-week-old sex-matched CD1 (Swiss) mice, obtained from the Laboratory Animal Services breeding facility of the University of Adelaide, were used in all experiments. The Animal Ethics Committee of The University of Adelaide approved all animal experiments (approval numbers S-2010-001 and S-2013-053). The study was conducted in compliance with the Australian Code of Practice for the Care and Use of Animals for Scientific Purposes (7th Edition 2004) and the South Australian Animal Welfare Act 1985.

### Bacterial strains and growth conditions

The pneumococcal strains used in this study are serotype 4 (WCH43), serotype 6A (WCH16), and their otherwise isogenic mutant derivatives (Table 1). Serotype-specific capsule production was confirmed by Quellung reaction, as described previously [25]. Opaque-phase variants of the three strains, selected on Todd-Hewitt broth supplemented with 1% yeast extract (THY)-catalase plates [26], were used in all animal experiments. Before infection, the bacteria were grown statically at 37°C in serum broth (10% heat-inactivated horse serum in nutrient broth) to *A*_600_ of 0.16 (equivalent to approx. 5 × 10^7^ CFU/ml).

**Table 1:** *S*. *pneumoniae* strains and primers used in this study.

Strain/Primer	Description (Sequence type)	Source/Reference
WCH16	Capsular serotype 6A clinical isolate (4966)	Women’s and Children’s Hospital, North Adelaide, Australia
WCH43	Capsular serotype 4 clinical isolate (205)	Women’s and Children’s Hospital, North Adelaide, Australia
WCH16::Δ*prtA*	*prtA* deletion mutant of WCH16 [Ery^R^]	Present study
WCH16::Δ*sodA*	*sodA* deletion mutant of WCH16 [Ery^R^]	Present study
WCH16::Δ*vanZ*	*vanZ* deletion mutant of WCH16 [Spec^R^]	Present study
WCH43::Δ*prtA*	*prtA* deletion mutant of WCH43 [Ery^R^]	Present study
WCH43::Δ*sodA*	*sodA* deletion mutant of WCH43 [Ery^R^]	Present study
WCH43::Δ*ulaA*	*ulaA* deletion mutant of WCH43 [Spec^R^]	Present study
WCH43::Δ*vanZ*	*vanZ* deletion mutant of WCH43 [Spec^R^]	Present study
*prtA* Ery X	5’-TTGTTCATGTAATCACTCCTTCTATTTATATAACTTCCAATAGATA-3’	
*prtA* Ery Y	5’-CGGGAGGAAATAATTCTATGAGTATAGAAAAAAATGGTTTATGTACTGA-3’	
*prtA* Flank F	5’-GAATGCATCTGATTTTTATCAGAC-3’	
*prtA* Flank R	5’-TCTAAAACCTCTTTGTTTACGAGAG-3’	
*prtA* UpSeq	5’-GAGCTTGGTTCCAAGTGGTTGATT-3’	
*sodA* Ery X	5’-TTGTTCATGTAATCACTCCTTCTTTCTTTCTATATGAAAATGATAACGC-3’	
*sodA* Ery Y	5’-CGGGAGGAAATAATTCTATGAGGAGGGAAGAATTGTTCTTCTCTTTTTAG-3’	
*sodA* Flank F	5’-CTTTGCGGATGAGAAAATCGTGAT-3’	
*sodA* Flank R	5’-GACAGATAAACCATAGTGTTGACGC-3’	
*sodA* UpSeq	5’-GCCAATGTTCACGCCTTTTATCAAC-3’	
*ulaA* Spec X	5’-TATGTATTCATATATATCCTCCTCTTGAATTGTTTTTGTAAGTTTATTATATA-3’	
*ulaA* Spec Y	5’-AAATAACAGATTGAAGAAGGTATAATATCTAGAAAAGGAGAAATAAAATGGTT-3’	
*ulaA* Flank F	5’-GCTATTAAAAAAATAGAGGAAGAAGGT-3’	
*ulaA* Flank R	5’-GCTGGATCCACAGCCTCTGTAATTC-3’	
*ulaA* UpSeq	5’-TCTTTGCAGTTTATGCGCCAGGTG-3’	
*vanZ* Spec X	5’-TATGTATTCATATATATCCTCCTCGTTTGAAGCCGTCTTCAACAAACA-3’	
*vanZ* Spec Y	5’-AAATAACAGATTGAAGAAGGTATAACTAATGATTAAAAAGGAGAATATAATG-3’	
*vanZ* Flank F	5’-CTTAAGGAAGTTCTACTTGAGCCG-3’	
*vanZ* Flank R	5’-GAAAACGCCGTGCATCTTCTCAGC-3’	
*vanZ* UpSeq	5’-CAAGACTGGGGTTAAAGAACCCGT-3’	

### Intranasal challenge of mice and extraction of total pneumococcal RNA

The protocols used for mouse challenge and analysis of *in vivo* gene expression by WCH16 and WCH43 have been described in detail previously [22, 23]. Briefly, groups of female mice were challenged intranasally (i.n.) with either WCH16 or WCH43 under anaesthesia. For the current study, RNA was extracted from pneumococci harvested from the nasopharynx and blood of at least 12 mice at each of 48, 72 and 96 h post-challenge using acid-phenol–chloroform–isoamyl alcohol (125:24:1; pH 4.5; Ambion) and purified using RNeasy minikit (Qiagen). The experiment was performed three times for each strain.

### Transcriptomic analyses

Microarray experiments were performed on whole genome *S*. *pneumoniae* PCR arrays obtained from the Bacterial Microarray Group at St George's Hospital Medical School, London (http://bugs.sghms.ac.uk/). The array was designed using TIGR4 base strain annotation [27] and extra target genes from strain R6 [28]. The array design is available in BμG@Sbase (Accession No. A-BUGS-14; http://bugs.sgul.ac.uk/A-BUGS-14) and also GEO (Platform GPL4001). Pair-wise comparisons were made between the nasopharynx and blood RNA samples from the 48, 72 and 96 h time points. RNA samples were reverse-transcribed using Superscript III (Invitrogen), labeled with either Alexa Fluor 546 or Alexa Fluor 647 dye using the 3DNA Array 900 MPX labeling kit (Genisphere) and then hybridized to the surface of the microarray, essentially as described [29, 30]. Slides were scanned at 10 μm resolution using a Genepix 4000B Scanner (Molecular Devices, USA) and spots were analyzed using the Spot plugin (CSIRO, Australia) within the R statistical software package (http://www.R-project.org). The Limma plugin for R [31] was used for data processing and statistical analysis and ratio values were normalized using the print-tip Loess normalization routine [32]. The replicate arrays were normalized to each other to give similar ranges of mRNA expression values. These statistics were used to rank the mRNAs from those most likely to be differentially expressed to the least likely using false-discovery rate values of *p*< 0.05. A two-sample Bayesian *t*-test was also used to analyze the transcriptomic data [33] using FlexArray software (McGill University, Canada), and values with a *p* = 0.05 were considered to be statistically significant. In this manner, microarray analysis examining RNA from infected nasopharynx vs blood was performed on at least 9 independent hybridizations for each strain, including at least one dye reversal per comparison.

### Gene Ontology (GO) classification and GO-based network construction

To gain a detailed view and a better understanding of the functions of the differentially regulated pneumococcal genes in the nasopharynx and blood, we carried out gene ontology (GO) classification and network analysis of the genes using our recently developed comparative GO web application [34]. Particular attention was paid to “molecular function” and “biological process” GO categories. Data from GO protein distributions were analyzed by two-sample Kolmogorov–Smirnov (K–S) and Goodness-of-Fit (Chi-square) tests [35]. We also utilized the information from the GO analysis for selection of genes subjected to mutation.

### Construction of mutants and assessment of bacterial growth *in vitro*

Defined, non-polar mutants of selected genes of interest were constructed in strains WCH16 and WCH43. Selection criteria included known or putative contribution to pneumococcal metabolism, pathogenesis and virulence. Mutants were constructed by overlap extension PCR as described previously [36] and validated by PCR and sequencing to be in-frame deletion mutation replacements. All PCR procedures were performed with the Phusion High Fidelity Kit (FINNZYMES). The primer pairs used for construction and validation of the mutants are listed in Table 1. In order to evaluate the growth rate of the mutants in comparison to the wild type, bacterial strains were grown in serum broth (SB) and *A*_600_ monitored overnight on a Spectramax M2 spectrophotometer (Millennium Science).

### Virulence assessment of mutants

To assess the virulence potential of mutants, groups of 12 male mice were challenged i.n. with approx 1 × 10^7^ CFU of either mutant or wild type bacteria in 50 μl SB, under deep anesthesia. The survival of mice (time to moribund) was monitored closely (four times daily for the first 5 days (and more frequently if they show signs of distress as outlined below), twice daily for the next 5 days, and then daily until 14 days after challenge. During the monitoring period, the condition of each mouse was measured based on a predetermined Clinical Record Score and written on a Clinical Record Sheet approved by the Animal Ethics Committee of The University of Adelaide. Mice are humanely euthanized either by CO_2_ asphyxiation or by cervical dislocation if they become moribund or show any evidence of distress. The following criteria were considered sufficient evidence of distress to warrant such intervention in order to minimize pain or suffering to the animals: loss of balance, extreme hyperactivity or other evidence of meningitis or CNS involvement; severe weight loss (>20% body weight); ear temperature falling below 24°C; paralysis or extreme reluctance or inability to move freely, and/or refusal or inability to eat or drink. Differences in survival times for mice between wild-type and mutants were analyzed by the Kaplan-Meier survival (log-rank [Mantel-Cox] and Gehan-Breslow-Wilcoxon) tests using GraphPad Prism v6 software.

### Pathogenesis experiments

For pathogenesis experiments, *S*. *pneumoniae* derivatives with mutations in genes of interest (Δ*prtA*, Δ*sodA* and Δ*vanZ*) and the isogenic wild-type strain (WCH16 or WCH43) were grown separately in SB to *A*_600_ = 0.16 (approx. 5 × 10^7^ CFU/ml). For this analysis, groups of 6 mice were anesthetized by intraperitoneal injection of pentobarbital sodium (Nembutal; Ilium) at a dose of 66 mg per g of body weight and separately challenged i.n. with 50 μl suspension containing approx. 3 × 10^6^ CFU of either wild-type or the isogenic mutant. At 48 h post-challenge, mice from each separate infection experiment were sacrificed and bacteria were enumerated from the nasopharynx, lungs and blood, as described previously [22, 23]. The experiment was repeated once.

## Results

In this work, we carried out a direct comparison of pneumococcal gene expression between the nasopharynx and blood of mice in order to identify differentially expressed genes between the two niches. The analyses were performed using existing transcriptomic data obtained from two *S*. *pneumoniae* strains, WCH16 and WCH43, after intranasal challenge of mice.

### Identification of differentially expressed genes between the nasopharynx and blood

**Fig 1:**
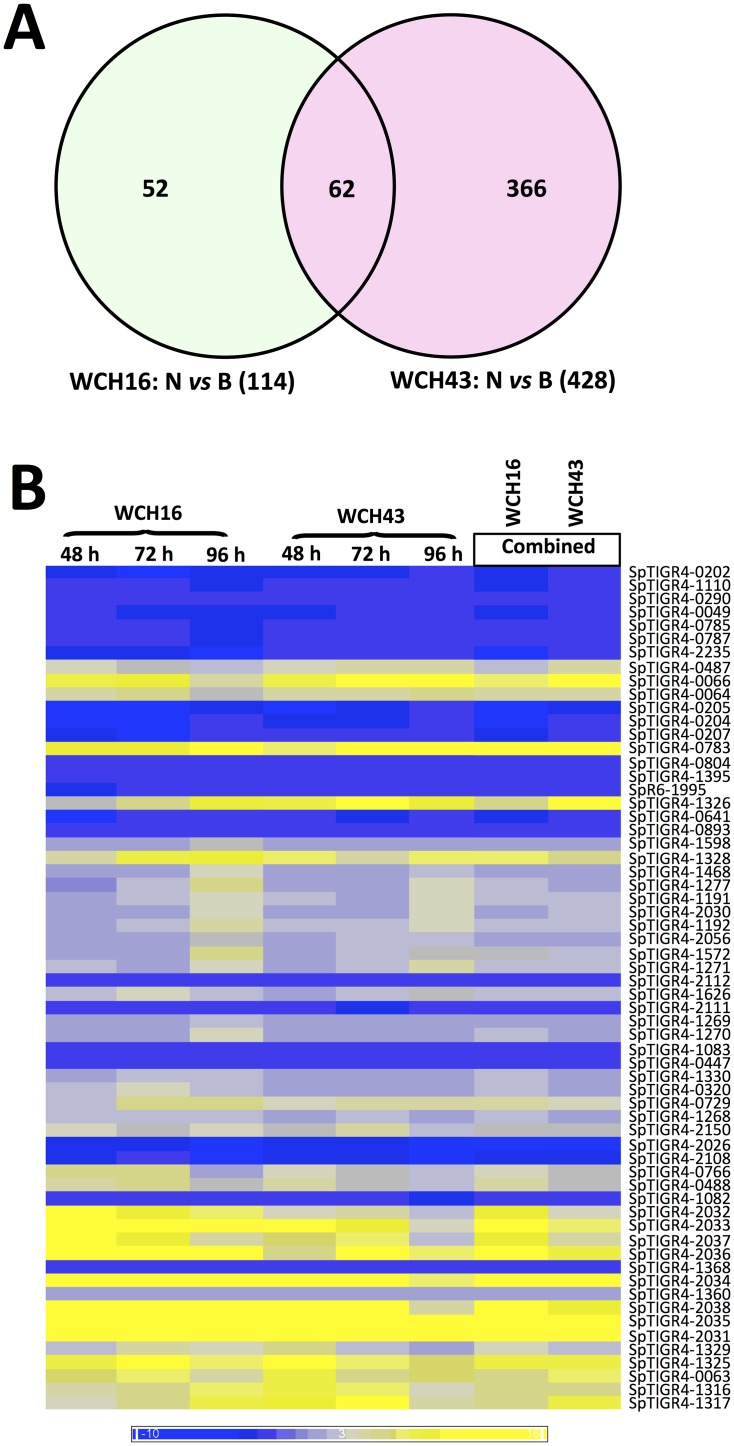
Regulation of pneumococcal gene expression between nasopharynx and blood of mice infected i.n. with WCH16 and WCH43. **(A)**. Venn diagram of differentially expressed genes from microarray comparisons of Nasopharynx (N) versus Blood (B) in WCH16 and WCH43. **(B).** Heat map showing regulated gene expression; yellow to blue scales represent fold difference in mRNA level; yellow, up-regulation in nasopharynx relative to blood; blue, downregulation in nasopharynx relative to blood.

We carried out a two-color microarray comparison of mRNA extracted from pneumococci harvested from the nasopharnyx and blood of mice at 48, 72 and 96 h post-infection in separate challenge experiments with either WCH16 or WCH43. The comparisons yielded 114 differentially regulated genes in the blood relative to the nasopharynx for WCH16, and 428 genes differentially regulated in the blood for WCH43, of which 62 differentially expressed genes were found to be common to both strains (Fig 1A and 1B). Details of combined expression values for all the 62 genes are in S1 Table. Fully annotated microarray data have been deposited (GEO accession number GSE73217).

### Functional classification of differentially expressed genes

**Fig 2:**
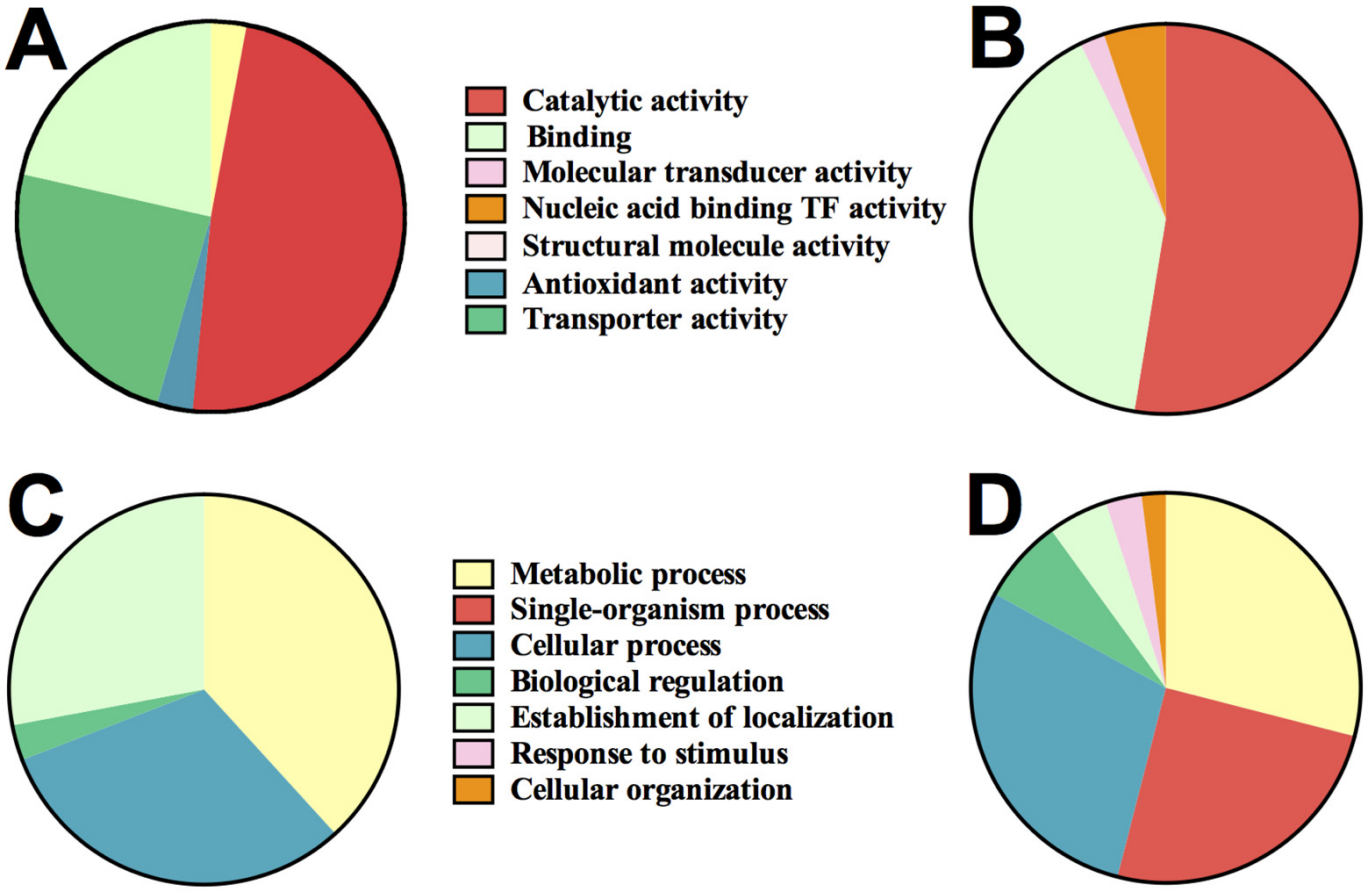
GO classification of pneumococcal genes differentially up-regulated in the nasopharynx and blood of mice. Molecular functional categories of genes (A), up-regulated in the nasopharynx, and (B), up-regulated in the blood. (C and D), Biological process categories of up-regulated genes in the nasopharynx (C), and up-regulated genes in the blood (D).

We utilized our recently developed comparative GO web application [34] to assess any differences in the functional categories of genes up-regulated either in the nasopharynx or blood, with particular attention to molecular function and biological process. The analyses showed that under “molecular function”, the GO protein distributions between the nasopharynx and blood were significantly different (*p* = 0.0008; two-sample K-S test). GO groups involved in transporter activity were significantly up-regulated in the nasopharynx relative to blood [*p* = 0.01; Chi square test], while those involved in transcription factor (DNA binding) activity were substantially up-regulated in the blood (Fig 2A and 2B). Similarly, under “biological process”, the GO protein distributions between the two niches were significantly different [*p* = 0.0043; two-sample K-S test] (Fig 2C and 2D), although this did not reach statistical significance for any of the GO groups identified between the two niches by Chi square test.

### Contribution of up-regulated pneumococcal genes to colonization and virulence

Earlier studies had shown that the pneumococcus undergoes spontaneous phase variation between a transparent and an opaque colony phenotype *in vitro*, with the transparent phenotype commonly associated with nasopharyngeal colonization and the opaque variant being favored in the blood [26, 37]. These findings led to the suggestion that phase variation might provide important clues to the interaction of the pneumococcus with its host, the opaque phenotype being significantly more virulent than the transparent phenotype [26, 38]. Interestingly, subsequent work in our laboratory indicated that niche-specific differences in expression of selected pneumococcal virulence genes were not attributable to phase variation. In order to eliminate the contribution of phase variation to selection of genes for further analysis, we initially compared gene expression of transparent pneumococci with those harvested from the nasopharynx, and also compared gene expression of opaque pneumococci with those harvested from the blood. We reasoned that such analyses would identify genes that are most likely to be essential for either colonization, or important for blood invasion or systemic disease. We also examined our existing *in vivo* transcriptomic comparisons of nasopharynx vs lungs, as well as data from lungs vs blood comparisons to guide our gene selection. In this manner, only pneumococcal genes that were either up-regulated in the nasopharynx or blood were selected for further analyses, independent of colony phenotype.

We then set an arbitrary threshold of >2-fold regulation to select differentially regulated genes in the two niches that are considered to be physiologically relevant. Consequently, 2 of the up-regulated genes in the nasopharynx (SP_0766 [*sodA*] and SP_2038 [*ulaA*]) and 2 genes up-regulated in the blood (SP_0049 [*vanZ*] and SP_0641 [*prtA*]) were selected for further analysis and validated by RT-PCR for both strains (Table 2). *S*. *pneumoniae* WCH43 derivatives with marked mutations in the 4 selected genes were constructed by targeted mutagenesis using overlap PCR. The *in vitro* growth rate of WCH16 and all its isogenic mutants was similar over a 3-hour period as well as overnight as judged by *A*_600_ measurements in serum broth on a Spectramax M2 spectrophotometer (Millennium Science). For WCH43, growth of the *sodA* mutant was slightly delayed (approx. 20 minutes) over the 3-hour growth period, as well as during overnight growth. Nevertheless, this delay in growth of the *sodA* mutant of WCH43 would not account for its attenuation in mice, as similar result were obtained for the *sodA* mutant of WCH16, as described below.

**Table 2:** Differential expression profiles of selected *S*. *pneumoniae* WCH16 and WCH43 genes between Nasopharynx and Blood by microarray analysis.

Gene ID^a^	Gene annotation	Fold change (Nasopharynx/Blood)	
		WCH16	WCH43
SP_0049	VanZ protein, putative	-2.61 (-59.03)^b^	-2.02 (-13.78)
SP_0641	Serine protease (PrtA)	-2.45 (-34.80)	-2.25 (-6.82)
SP_0766	Superoxide dismutase, manganese-dependent (SodA)	4.16 (7.15)	3.32 (3.39)
SP_2038	PTS system ascorbate-specific transporter subunit IIC (UlaA)	15.48 (22.94)	9.66 (278.20)

^**a**^ Gene IDs were obtained from the genome of *S*. *pneumoniae* TIGR4 (serotype 4) as deposited in the Kyoto Encyclopedia of Genes and Genomes (KEGG) database.

^**b**^ Data in parentheses represent corresponding real time RT-PCR expression values from comparisons of total mRNA from at least 2 independent experiments.

**Fig 3:**
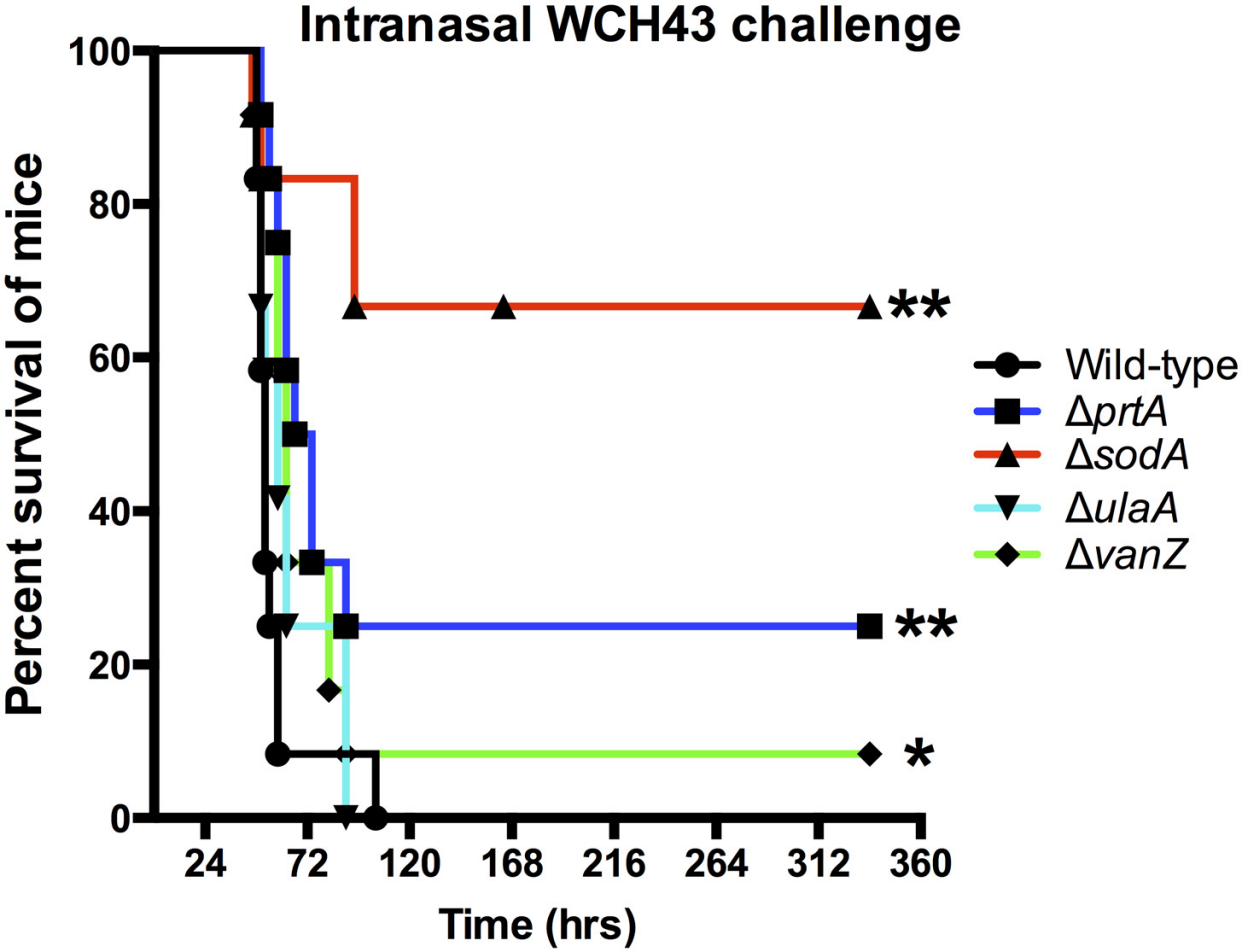
Survival times for mice after i.n. challenge with WCH43 and isogenic mutant derivatives. Groups of 12 CD1 male mice were challenged i.n. with approx. 1 × 10^7^ CFU of the indicated strains. Survival curves were compared using log-rank [Mantel-Cox] and Gehan-Breslow-Wilcoxon) tests. (* *P*<0.05; ** *P*<0.01).

**Fig 4:**
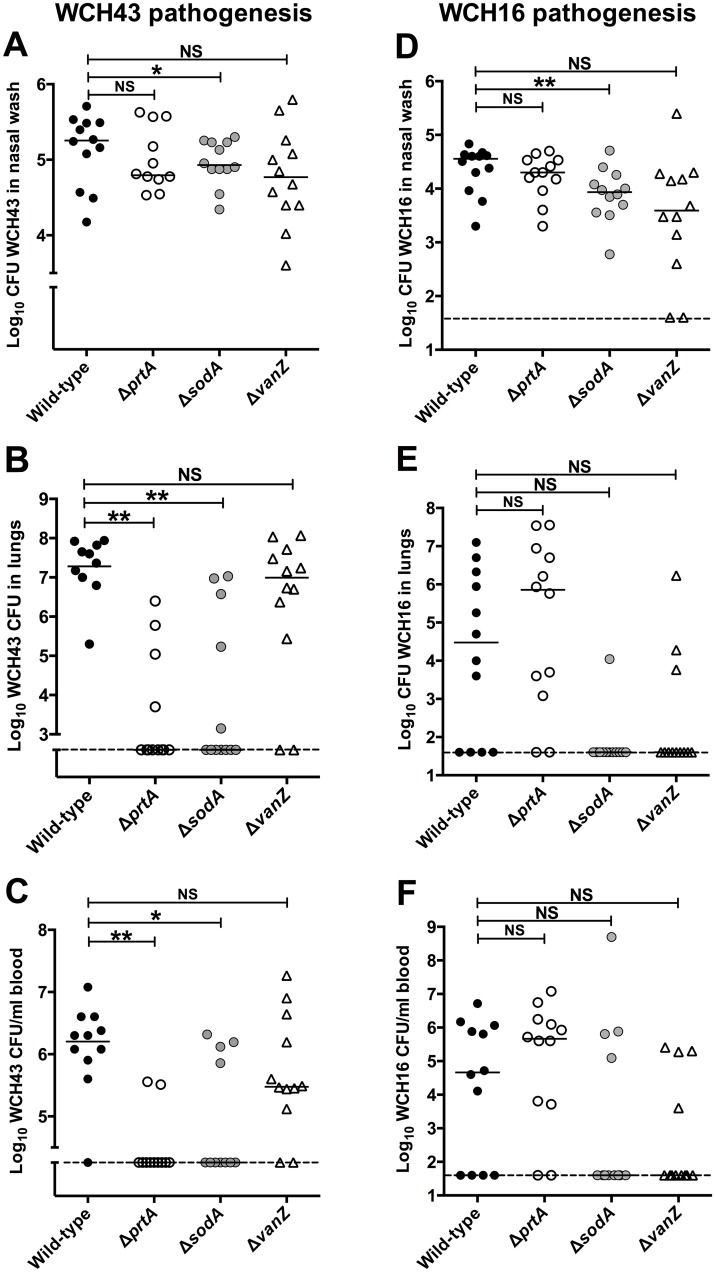
Pathogenesis of WCH43 (A-C) and WCH16 (D-F) and their isogenic mutant derivatives in male CD1 mice. Bacteria were enumerated from the nasopharynx (A, D); lungs (B, E) and blood (C, F) of each mouse at 48 h post-infection (n = 12 per group per time-point). Horizontal line represents geometric mean; horizontal broken line denote limit of detection (40 CFU). *, *P* < 0.05; ** *P*<0.01; unpaired *t*-test, one-tailed.

After intranasal challenge of mice, the Δ*prtA*, Δ*sodA* mutants and, to a lesser extent, the Δ*vanZ* mutant of WCH43 were significantly attenuated for virulence, while the Δ*ulaA* mutant was essentially as virulent as the wild type (Fig 3). To assess the involvement of *prtA*, *sodA* and *vanZ* in colonization or blood invasion, we challenged groups of mice i.n. with approx. 3 × 10^6^ CFU of either mutant or the isogenic wild-type WCH16 or WCH43 strain and harvested bacteria from the nasopharynx, lungs and blood at 48 h post-infection. For WCH43, we found that only the Δ*sodA* mutant was significantly attenuated for colonization of the nasopharynx (Fig 4A), while the numbers of Δ*prtA* and Δ*sodA* mutants were significantly lower in lungs (Fig 4B) and blood (Fig 4C) compared to wild type. However, while the numbers of the Δ*vanZ* mutant bacteria were generally lower in blood than those for the wild type, this did not reach statistical significance. To verify these results, we repeated the pathogenesis experiment using WCH16 and its isogenic Δ*prtA*, Δ*sodA* and Δ*vanZ* mutants. As observed for WCH43, the Δ*sodA* mutant was also significantly attenuated for colonization of the nasopharynx (Fig 4D), However, the differences in pathogenesis of the mutants in WCH16 in lungs (Fig 4E) and blood (Fig 4F) did not reach statistical significance, consistent with our previous observations that WCH16 displays minimal lung and blood involvement during pathogenesis [39, 40].

## Discussion

In spite of the availability of antimicrobial therapy and conjugate vaccines, *S*. *pneumoniae* continues to be responsible for massive global morbidity and mortality. The problems associated with pneumococcal disease are largely due to our incomplete understanding of the molecular mechanisms underlying pneumococcal pathogenesis. Therefore, successful control of pneumococcal disease and identification of novel vaccine candidates will require a detailed understanding of the molecular mechanisms and virulence determinants underpinning the development of invasive pneumococcal disease (IPD). Such investigations will include a thorough examination of factors up-regulated during transition from nasopharyngeal colonization to invasive or meningeal infection, in conjunction with developing an understanding of the regulatory mechanisms underpinning such translocation.

As an extension of our efforts toward identifying the critical determinants of pathogenesis, we have used microarray analysis to do a direct comparison of pneumococcal gene expression between the nasopharynx and blood of mice. This assessment was undertaken in order to identify the genes specifically up-regulated during blood invasion from the nasopharynx (“occult bacteremia”), which is often a complication of pneumococcal carriage in young children [24, 41]. This is critical in the context of optimization of a multi-component protein vaccine that targets all forms of pneumococcal disease, considering the hypothesis that many pneumococcal virulence factors have dual roles in the maintenance of carriage and invasive disease [38, 42, 43].

Our analysis identified a number of common factors significantly up-regulated in the nasopharynx relative to blood in the two pneumococcal strains; not surprisingly, the vast majority of the encoded proteins function in carbohydrate metabolism and/or transporter activity. Mutation of one of the two genes up-regulated in the nasopharynx, *sodA*, resulted in significant attenuation of virulence in a mouse intranasal challenge model, in agreement with previous reports [42, 44]. In a pathogenesis experiment, there was significant reduction in numbers of *sodA* mutant bacteria recovered from the nasopharynx and a concomitant significant reduction in the lungs and blood at 48 hr post-challenge. These results are consistent with a significant role for SodA in oxidative stress management [44, 45], suggesting it might be a good a drug target against carriage. However, deletion of *ulaA* (with a putative function of the encoded product in transporter activity) did not have any effect on virulence, suggesting functional redundancy.

We also analyzed the genes that were significantly up-regulated in the blood relative to nasopharynx in the two pneumococcal strains. Most of the genes encode enzymes involved in metabolism of cofactors, vitamins and amino acids, as well as those required for transcriptional regulation of metabolites. This would be consistent with the need to up-regulate such factors for sensing and adaptation to challenging environments such as the blood. For some of these factors, their role in pneumococcal virulence is well documented. For instance, CbpA (SpR_1995/SP_2190) has been shown to be involved in multiple stages of pneumococcal pathogenesis [43, 46–48]. Another protein, MalX (SP_2108), was shown to be important for colonization [49] and lung infection [42, 44]. Like CbpA, the finding in this study that *malX* is significantly up-regulated in the blood relative to the nasopharynx suggests that it might also be involved in multiple stages of pathogenesis, which would make it an ideal protein vaccine candidate.

We then mutated 2 of the genes, *prtA* and *vanZ*, encoding a cell wall-localized subtilisin-like serine protease (PrtA) and a putative vancomycin resistant protein (VanZ), respectively. The *prtA* mutant was significantly attenuated in a mouse intranasal challenge model in the WCH43 background; the *vanZ* mutant also exhibited a small, but statistically significant attenuation. The attenuation of the *prtA* mutant is in agreement with a previous report [50], although in that study, the challenge was via an intraperitoneal route, using a different pneumococcal strain, D39 [50]. Interestingly, in our study, pathogenesis studies show significantly low numbers of WCH43 *prtA* mutant bacteria in the bloodstream of intranasally-challenged mice relative to wild-type bacteria, which would be consistent with its attenuated virulence, but in contrast to the earlier study which showed more *prtA* mutant bacteria in the blood of intraperitoneally challenged mice [50]. Another recent study showed modest effects of a D39Δ*prtA* mutation in lungs and blood of intranasally-infected mice [51]. A possible explanation for the apparent contradiction in these results could be that expression of PrtA is more important for pathogenesis and virulence of WCH43, while this effect is masked by other virulence factors produced by D39 (which displays high grade bacteremia), suggesting strain-specific differences. This is further highlighted by our pathogenesis results with WCH16 *prtA* mutant. Moreover, the different routes of infection and challenge doses used in these studies could also account for the discordant results. The discrepancy in attenuation of virulence of a specific factor in different pneumococcal backgrounds is not unprecedented, as was reported for *cbpA* ^*-*^ [*pspC* ^-^] [25, 43, 47, 52, 53] and *psaR* [54] mutants. With respect to VanZ, its specific role in pneumococcal pathogenesis is yet to be described, although it was found to be essential for lung infection [42] and its homolog has been shown to confer resistance to teicoplanin in *Enterococcus faecium* [55].

It is as yet unclear how PrtA contributes to the virulence of *S*. *pneumoniae*. It was shown previously that PrtA acts by cleaving apolactoferrin (the iron-free form of human lactoferrin) to produce a lactoferricin-like peptide, which is highly bactericidal for pneumococci [56]. This would suggest that expressing PrtA might be counter-productive, because anti-PrtA antibodies could inhibit killing of the pneumococcus. Nevertheless, strongly reactive anti-PrtA antibody was prominent in convalescent-phase human sera [50, 57, 58], and a *prtA* mutant was attenuated for virulence as demonstrated in the present study. Therefore, more work, both *in vitro* and *in vivo*, is needed to clarify the role of PrtA in the biology of *S*. *pneumoniae*, and to determine whether it is worthy of further consideration as a vaccine candidate.

Other workers have compared transcriptomes of pneumococci harvested from various *in vitro* and *in vivo* sources by microarray analysis or RNA-Seq. For example, one previous study [59] used *in vitro* alternatives for specific *in vivo* niches (e.g. co-culture with Detroit 562 cells to mimic nasopharyngeal colonization; co-culture with type II pneumocytes to mimic lung infection). The workers further compared whole transcriptomes of a particular pneumococcal strain (TIGR4) harvested *in vitro* (to mimic colonization) with whole transcriptomes of a different pneumococcal strain (D39) harvested from mouse blood (to mimic invasive disease). Recently, another group of workers [60] characterized pneumococcal RNA harvested from a human respiratory epithelial cell line (to mimic lung infection), using *S*. *pneumoniae* EF3030, which causes focal pneumonia in their infection experiments. Furthermore, Pettigrew et al. compared transcriptional profiles of planktonic and biofilm-grown EF3030 with influenza A virus-dispersed bacteria. Genes that have been well characterized to play a prominent role in pneumococcal pathogenesis and/or virulence (such as *ply*, *pspA*, *cbpA*, *malX*) were commonly identified in those previous studies and in our study. Nevertheless, we are of the view that our mouse intranasal infection model closely mimics natural progression of invasive pneumococcal disease in humans; the use of multiple strains of different pathogenicity characteristics also increases the likelihood of identifying common, novel genes that might be critical to pneumococcal pathogenesis.

## Supporting Information

S1 Table Differential gene expression profiles of *S*. *pneumoniae* WCH16 and WCH43 between Nasopharynx and Blood by microarray analysis. (DOCX)
