# Supplementary material for: Characterization of Pneumococcal Genes Involved in Bloodstream Invasion in a Mouse Model
Source: PLoS One. 2015 Nov 5;10(11):e0141816. doi: 10.1371/journal.pone.0141816 (PMC4634996; doi:10.1371/journal.pone.0141816)
Supplement: S1 Table — (DOCX) [file pone.0141816.s001.docx]

**S1 Table. Differential gene expression profiles of *S. pneumoniae* WCH16 and WCH43 between Nasopharynx and Blood by microarray analysis.**

| **Gene ID (TIGR4/R6)^a^** | **Gene annotation** | **Fold change (Nasopharynx/Blood)** | |
| --- | --- | --- | --- |
|  |  | **WCH16** | **WCH43** |
| **SP_0049** | **VanZ protein, putative** | **-2.61 (-59.03)^b^** | **-2.02 (-13.78)** |
| SP_0063 | PTS system transporter subunit IID | 6.2 | 7.37 |
| SP_0064 | PTS system transporter subunit IIA | 4.58 | 4.92 |
| SP_0066 | Aldose 1-epimerase | 7.57 | 12.14 |
| SP_0202 | anaerobic ribonucleoside triphosphate reductase | -3.36 | -2.2 |
| SP_0204 | Acetyltransferase | -3.89 | -2.12 |
| SP_0205 | Anaerobic ribonucleoside-triphosphate reductase activating protein | -3.87 | -3.03 |
| SP_0207 | Phosphoribulokinase | -3.23 | -1.8 |
| SP_0290 | Dihydrofolate synthetase | -1.93 | -1.64 |
| SP_0320 | Gluconate 5-dehydrogenase | 3.02 | 1.72 |
| SP_0447 | Ilv (Valine, leucine and isoleucine biosynthesis) | -1.49 | -1.89 |
| SP_0487 | Hypothetical protein | 3.11 | 4.66 |
| SP_0488 | Hypothetical protein | 4.6 | 3.51 |
| **SP_0641** | **Serine protease (PrtA)** | **-2.45 (-34.80)** | **-2.25 (-6.82)** |
| SP_0729 | Cation transporter E1-E2 family ATPase | 4.8 | 4.44 |
| **SP_0766** | **Superoxide dismutase, manganese-dependent (SodA)** | **4.16 (7.15)** | **3.32 (3.39)** |
| SP_0783 | Biotin synthase | 10.3 | 11.48 |
| SP_0785 | HlyD family secretion protein | -2.28 | -1.89 |
| SP_0787 | Putative ABC transport system permease protein | -2.36 | -1.48 |
| SP_0804 | 4-methyl-5 (b-hydroxyethyl)-thiazole monophosphate biosynthesis protein | -1.85 | -1.74 |
| SP_0893 | Transcriptional repressor, arginine metabolism | -1.99 | -1.77 |
| SP_1082 | Acetyltransferase | -1.69 | -2.12 |
| SP_1083 | Putative DNA-binding protein | -1.85 | -2.2 |
| SP_1110 | Bifunctional riboflavin kinase (RibC, RibF) | -2.48 | -1.94 |
| SP_1191 | Tagatose-6-phosphate kinase | 2.71 | 2.62 |
| SP_1192 | Galactose-6-phosphate isomerase subunit (LacB) | 2.82 | 3.1 |
| SP_1268 | LicB protein | 2.78 | 2.27 |
| SP_1269 | Choline kinase | 1.91 | 1.44 |
| SP_1270 | Zinc-containing alcohol dehydrogenase | 2.6 | 1.54 |
| SP_1271 | IspD | 3.1 | 3.01 |
| SP_1277 | 2-C-methyl-D-erythritol 4-phosphate cytidylyltransferase | 2.66 | 2.22 |
| SP_1316 | V-type ATP synthase subunit B | 6.05 | 6.43 |
| SP_1317 | V-type ATP synthase subunit A | 6.24 | 8.16 |
| SP_1325 | Gfo/Idh/MocA family oxidoreductase | 8.79 | 8.31 |
| SP_1326 | Neuraminidase C (NanC) | 6.09 | 10.27 |
| SP_1328 | Sodium:solute symporter family protein | 7.26 | 6.44 |
| SP_1329 | N-acetylneuraminate lyase | 3.97 | 3.16 |
| SP_1330 | N-acetylmannosamine-6-phosphate 2-epimerase (NanE) | 2.65 | 2.04 |
| SP_1360 | Homoserine kinase | 1.6 | 1.36 |
| SP_1368 | Psr protein (LytR family transcriptional regulator) | -1.86 | -2.01 |
| SP_1395 | PhoU (phosphate transport system regulatory protein) | -1.8 | -1.71 |
| SP_1468 | PdxS (pyridoxal biosynthesis lyase) | 2.09 | 1.8 |
| SP_1572 | Non-heme iron-containing ferritin | 3.2 | 2.81 |
| SP_1598 | Phosphomethylpyrimidine kinase | 1.94 | 1.65 |
| SP_1626 | RpsO | 2.9 | 2.38 |
| SP_2026 | Bifunctional acetaldehyde-CoA/alcohol dehydrogenase (Adh2) | -4.93 | -4.26 |
| SP_2030 | Transketolase | 2.29 | 2.7 |
| SP_2031 | L-ascorbate 6-phosphate lactonase | 30.13 | 18.84 |
| SP_2032 | BglG family transcriptional regulator | 8.99 | 3.85 |
| SP_2033 | AraD | 17.88 | 7.63 |
| SP_2034 | L-xylulose 5-phosphate 3-epimerase | 26.03 | 13.87 |
| SP_2035 | UlaD | 20.29 | 14.67 |
| SP_2036 | PTS system transporter subunit IIA | 13.95 | 8.07 |
| SP_2037 | PTS system transporter subunit IIB | 9.14 | 5.13 |
| **SP_2038** | **PTS system ascorbate-specific transporter subunit IIC (UlaA)** | **15.48 (22.94)** | **9.66 (278.20)** |
| SP_2056 | N-acetylglucosamine-6-phosphate deacetylase | 2.16 | 2.09 |
| SP_2108 | Maltose/maltodextrin ABC transporter maltose/maltodextrin-binding protein (MalX) | -3.33 | -3.2 |
| SP_2111 | MalA protein | -1.63 | -1.91 |
| SP_2112 | Maltose operon transcriptional repressor (MalR) | -1.59 | -1.75 |
| SP_2150 | Ornithine carbamoyltransferase | 3.6 | 3.59 |
| SP_2190 (SpR6_1995) | Choline binding protein A (CbpA) | -1.91 | -1.51 |
| SP_2235 | Response regulator ComE | -3.95 | -1.76 |

**^a^** Gene IDs were obtained from the genomes of *S. pneumoniae* TIGR4 (serotype 4) and R6 (unencapsulated serotype 2 derivative) as deposited in the Kyoto Encyclopedia of Genes and Genomes (KEGG) database.

**^b^** Data in parentheses represent corresponding real time RT-PCR expression values from comparisons of total mRNA from at least 2 independent experiments.
